# A non-inferiority randomised controlled trial comparing self-instruction with instructor-led method in training of layperson cardiopulmonary resuscitation

**DOI:** 10.1038/s41598-020-79626-y

**Published:** 2021-01-13

**Authors:** Ying-Chih Ko, Chih-Wei Yang, Hao-Yang Lin, Wen-Chu Chiang, Ming-Ju Hsieh, Matthew Huei-Ming Ma

**Affiliations:** 1 Department of Emergency Medicine, National Taiwan University Hospital, No. 7 Chung Shan South Rd., Taipei, 100 Taiwan; 2 Department of Medical Education, National Taiwan University Hospital, Taipei, Taiwan; 3 Department and Graduate Institute of Medical Education and Bioethics, College of Medicine, National Taiwan University, Taipei, Taiwan; 4 Department of Emergency Medicine, National Taiwan University Hospital Yun-Lin Branch, Yun-Lin County, Taiwan

**Keywords:** Cardiology, Health care

## Abstract

Our study aimed to compare the effect of self-instruction with manikin feedback to that of instructor-led method on cardiopulmonary resuscitation (CPR) and automated external defibrillator (AED) skill performance. In our randomized non-inferiority trial, 64 non-healthcare providers were randomly allocated into self-instruction and instructor-led groups. Both groups watched a 27-min standardized teaching video. Participants in the self-instruction group then performed hands-on practice on the Resusci Anne QCPR with a device-driven feedback, while those in the instructor-led group practiced manikins; feedback was provided and student’s questions were answered by instructors. Outcomes were measured by blinded evaluators and SkillReporter software. The primary outcome was the pass rate. Secondary outcomes were scores of the knowledge test and items of individual skill performance. The baseline characteristics of the two groups were similar. The pass rates were 93.8% in both group (absolute difference 0%, *p* = 0.049 for noninferiority). The knowledge test scores were not significantly different. However, the self-instruction group performed better in some chest compression and ventilation skills, but performed worse in confirming environmental safety and checking normal breathing. There was no difference in AED skills between the two groups. Our results showed the self-instruction method is not inferior to the instructor-led method.

## Introduction

It has been proven that patients with cardiac arrest have higher survival rate if they receive bystander cardiopulmonary resuscitation (CPR) before prehospital personnel arrived^1^. Besides, patients who received bystander CPR and defibrillation had lower risk of brain damage or nursing home admission and death from any cause^2^. In the last few years, an increasing percentage of out-of-hospital cardiac arrest (OHCA) victims received bystander CPR^3–5^. For further increasing rates of bystander CPR, many interventions, including dispatcher-assisted bystander CPR, were performed^6^. One study showed high-quality CPR was associated with improved outcomes^7^. Therefore, it is also important to perform high-quality CPR for patients with cardiac arrest. The International Liaison Committee on Resuscitation (ILCOR) suggested that educational efficiency is one of three multiplicands that would affect the survival^8,9^. Enabling more citizens to perform high-quality CPR through more efficient educational methods helps the survival of patients with OHCA^10^.

To increase the number of citizens who know how to perform CPR, many instructor-led basic life support (BLS) courses are held. However, this kind of course has some disadvantages. Some surveys showed the first two reasons why students did not take BLS courses were no time for participation and inconvenience^11,12^. In addition, some students reported that they have learning and performance anxiety in an unfamiliar setting. Some learners also felt that they had no enough time to practice on manikins. Thus, they were not so familiar with CPR skills^13^. In addition, an inconsistency in instruction was also found in instructor-led BLS courses^14^.

Therefore, self-instruction courses have been developed to improve above disadvantages of traditional CPR courses. Such self-instruction courses, which did not require instructors, usually include game program on a website, a short video, and manikin with or without automated feedback for learners to perform hands-on practice. Such teaching method enables mass training because training capacity gets improved^15^. It revealed that the subjects with digital CPR training had better knowledge scores, but poorer skill performances of chest compression rates, compared to those with traditional training in one meta-analysis^15^. Nevertheless, the efficacy of self-instruction on resuscitation training and automated external defibrillator (AED) skill performance in Taiwan remains unclear. Digital teaching materials, such as videos or websites, have their own advantages because of accessibility and repeatability; however, the best way to teaching BLS courses is still undetermined. In addition, although many studies had been conducted to compare the efficacy between the two methods, the results remained controversial^16–20^.

Therefore, this study aimed to compare the efficacy of self-instruction with that of instructor-led method on laypersons who were taking BLS courses in an educational setting.

## Methods

### Trial design

We performed a randomized non-inferiority trial after it received approval from the institutional review board of the National Taiwan University Hospital (NTUH) and was reported following the Consolidated Standards of Reporting Trials 2010 statement^21^. All methods were carried out in accordance with relevant guidelines and regulations.

### Participants

We invited administrative clerks and other non-health care providers who worked in the NTUH to join our study by using e-mail and poster. The NTUH is a tertiary teaching hospital with 2,450 beds in Taipei City, Taiwan. In the e-mail, they were instructed to contact the study assistants if they were interested in or wished to join the study. Informed consent from the participants was then obtained after the questions arising from the participants were answered.

The inclusion criteria for the study participants were as follows: (1) participants over 18 years; and (2) those without prior CPR training, or whose last course was more than 2 years prior to the study. Those who were physically unsuited to performing CPR and AED were excluded from our study.

### Interventions

The participants were randomly assigned by using permuted block technique into two groups with the ratio of 1:1: the self-instruction group and instructor-led group. The participants were blinded to their assigned groups until they were contacted to join the course. In addition, they were also blinded to the purpose of the study until the end of the study.

Before joining the course, the participants in both groups were asked to watch a 27-min standardized teaching video on the website. The content of the teaching video included details regarding the basic BLS concept (1 min and 40 s), how to perform CPR (9 min and 50 s), how to use an AED (1 min and 40 s), the Heimlich manoeuvre (4 min and 50 s), a demonstration of performing CPR and the use of an AED (5 min), and paediatric BLS (4 min). The teaching video content followed the guidelines published in 2015^7,22,23^.

The course was held in the classroom of the clinical skill centre of the NTUH. On arrival at the classroom, the instructors in the instructor-led group had a discussion with the participants and answered the question about BLS as well as the video content arising from them. The self-learning group had no such discussion. Then, all the participants received a skill pre-test. The skills pre-test scenario required them to deal with a middle-aged man who had suddenly collapsed in front of them as they were walking along the street. Each participant was required to demonstrate how they would react to this scenario using the manikin. The duration of CPR was 2 min, which started from the first chest compression. After 2 min of CPR, an AED was given to the participant. The skill pre-test was completed when the participant resumed chest compressions after performing a shock with the AED or 2 min after acquiring an AED. All participants did not receive any kind of feedback on their performance in the pre-test.

After the skill pre-test, participants in the instructor-led group performed a 30-min hands-on practice on the Resusci Anne QCPR (Laerdal Company, Norway) with feedback from the instructors, and function of automated feedback was turned off in the instructor-led group. The instructors would give suggestions and adjust the skill performance based on the assessment in the checklist, including compression rate, compression depth, chest recoils and hand position throughout the whole period of hands-on practice. Also, the instructors would answer the questions raised by the participants. Each participant had practice at least three times. They could practice more if they still felt unfamiliar with the CPR skills, while no participant practiced more than five times. The total hands-on practice time in the instructor-led group took about half an hour. The ratio of instructor and student was 1:6, and the ratio of manikin to student was 1:3. The participants also practised AED skills on manikins.

In contrast, the participants in the self-instruction group performed hands-on practice on the same kind of manikin with automated feedback. The participants watched a screen situated alongside the manikin to acquire real-time feedback on the performance of the compression position, compression depth, compression rate, chest recoils, and ventilation volume, as they practised CPR. Each participant in the self-instruction group practised CPR and AED skills alone until they felt confident and familiar with the skills, or 10 min had passed. During hands-on practice, if the participants were unsure about how to perform some skills, the teaching video could be reviewed using one computer.

Immediately after training, both groups received written and skill tests. The written test comprised ten multiple-choice questions. The skill test after training was the same as that before hands-on practice.

### Outcomes

Two blinded assessors reviewed the recorded videos and evaluated the participants’ performance during the skill test using a checklist (Supplementary Table 1). The checklist included a list of skills required to perform CPR and AED as listed in the guidelines^7,22,23^. Before evaluating the skills performance, the assessors reached an agreement on how to evaluate the performance of each skill. The skill performance assessment was classified into three categories, including chest compressions, ventilation, and use of the AED. Each skill category was qualified if 60% of related checklist items met standards, and pass was defined as all three categories qualified. The performance of CPR during skill tests was also recorded by the Resusci Anne Wireless SkillReporter (Laerdal Medical, Stavanger, Norway).

**Table 1:** The characteristics of participants between two groups.

Characteristics	Self-instruction (n = 32)	Traditional instruction (n = 32)	*p*-value
Age (mean ± SD)	38.8 ± 9.4	41.3 ± 9.7	0.29
Male (%)	6 (9.4%)	7 (10.9%)	0.50
Height (cm)	160.3 ± 6.5	165.4 ± 8.5	0.009
Weight (kg)	57.4 ± 9.3	61.0 ± 12.3	0.19
CPR training before (years)	2.9 ± 0.8	2.8 ± 0.3	0.83

SD, standard deviation; CPR, cardiopulmonary resuscitation.

The primary outcome was the pass rate of the skill test at the course conclusion. The secondary outcomes were the scores of the knowledge test, and items of individual skill performance in the checklist and those recorded by the computers, such as mean compression rate, mean percentage of adequate compression rate, mean compression depth, mean percentage of complete recoil, and mean percentage of correct ventilation volume. The correct compression rate was defined as a compression rate of 100–120 cpm. The correct ventilation volume was defined as the ventilation volume between 400–700 mL.

### Sample size

With a power of 80%, a significance of 5%, an expected pass rate of traditional instruction of 95%, and a non-inferiority margin of 10%, we needed at least 29 patients in each group.

### Statistical analysis

The objective of the study was to show that the pass rate of the skill test in participants of the self-instruction group was not inferior to that of the instructor-led group. Six of the 18 items in the skill checklist contained three performance grades: performing the skill most (> 75%), often (25%–75%), and less (< 25%) of the time. We transformed the grades into 2, 1, and 0 before statistical analysis. The remaining items contained two grades: performing or not performing the skill. We transformed the grades into 1 and 0. The chi-square test and Student’s t-test were used to compare the differences between the two groups. Statistical analysis was performed using the SAS software (Version 9.4, SAS Institute Inc., Cary, NC, USA). A two-tailed *p* value of < 0.05 was considered statistically significant.

## Results

During the study period, a total number of 307 emails for invitation were sent and 65 participants were finally assessed for eligibility. After excluding one person physically unsuited to perform CPR, 64 participants were finally included, with 32 in the self-instruction group. All the participants completed the protocol. The characteristics of the study participants are shown in Table 1. Age, gender, weight, and prior CPR training experience were similar between the two groups, except that the participants in the instructor-led group were taller. Most of the participants were female.

In the primary outcome, the pass rates were the same between the two groups (93.8% vs. 93.8%) (Table 2). The proportion of the difference in passing the examination was 0%. The 95% confidence interval (CI) did not include an inferiority margin of − 10% (95% CI, 85.4% to 100%, *p* = 0.049). In the secondary outcomes, the knowledge test scores in both groups were not significantly different (self-instruction vs. instructor-led 9.5 vs. 9.6, *p* = 0.37) (Table 3). In the skill test, compared to the instructor-led group, the self-instruction group performed better in mean compression depth (48.4 vs. 42.0 mm, *p* = 0.002), mean percentage of full chest recoils (77.7% vs. 56.4%, *p* = 0.014), and mean percentage of correct ventilation volume (96.5% vs. 75.1%, *p* = 0.013), but performed worse in confirming environmental safety (47% vs. 78%, *p* = 0.010) and checking normal breathing < 10 s before starting CPR (26% vs. 97%, *p* < 0.001) (Tables 2, 4). The other items of individual skills were similar, including AED skills (Table 2).

**Table 2:** Scores of individual skill performance evaluated by evaluators with the checklist.

Individual skill item (mean ± SD)	Self-instruction (n = 32)	Traditional instruction (n = 32)	*p*-value
1. Makes sure safety of the environment	0.47 ± 0.51	0.78 ± 0.42	0.009
2. Checks consciousness	1.00 ± 0	1.00 ± 0	–
3. Checks breathing < 10 s	0.26 ± 0.45	0.97 ± 0.18	< 0.001
4. Calls for help	0.90 ± 0.30	0.91 ± 0.30	0.97
5. Corrects compression position	1.00 ± 0	0.97 ± 0.18	0.33
6. Corrects compression rate (100–120 bpm)	1.44 ± 0.84	1.84 ± 0.52	0.024
7. Corrects compression depth (5–6 cm)	1.66 ± 0.65	1.44 ± 0.76	0.22
8. Completes chest recoil	2.00 ± 0	2.00 ± 0	–
9. Opens airway	1.75 ± 0.57	1.97 ± 0.18	0.044
10. Chest elevation when giving breaths	1.59 ± 0.71	1.63 ± 0.75	0.87
11. Correct compression/breath ratio (30:2)	1.00 ± 0	1.00 ± 0	–
12. No unnecessary compression interruptions	2.00 ± 0	1.88 ± 0.34	0.044
13. Activates AED as soon as possible	0.56 ± 0.50	0.78 ± 0.42	0.06
14. Corrects AED pad position	0.81 ± 0.40	0.78 ± 0.42	0.76
15. Connects pad to AED machine	1.00 ± 0	1.00 ± 0	–
16. Clears site when analysing rhythm by AED	1.00 ± 0	1.00 ± 0	–
17. Clears site when defibrillation by AED	1.00 ± 0	1.00 ± 0	–
18. Resumes chest compression immediately after defibrillation	1.00 ± 0	1.00 ± 0	–
Pass rate, n (%)	30 (93.8%)	30 (93.8%)	

SD, standard deviation; bpm, beats per minute; AED, automated external defibrillator.

**Table 3:** The scores of knowledge test between groups.

	Self-instruction (n = 32)	Traditional instruction (n = 32)	*p*-value
CPR knowledge test score (mean ± SD) (full score 10)	9.5 ± 0.8	9.6 ± 0.6	0.37

CPR, cardiopulmonary resuscitation; SD, standard deviation.

**Table 4:** Individual skill performance recorded by the SkillReporter.

Individual skill item (mean ± SD)	Self-instruction (n = 32)	Traditional instruction (n = 32)	*p*-value
Mean compression depth (mm)	48.39 ± 6.87	41.97 ± 8.76	0.002
Mean percentage of correct compression rate (100–120 bpm) (%)	47.78 ± 35.34	50.96 ± 37.05	0.73
Mean compression rate (bpm)	120.71 ± 10.61	115.42 ± 11.86	0.07
Mean percentage of full chest recoil (%)	77.74 ± 28.59	56.35 ± 37.03	0.014
Mean percentage of correct hand position (%)	96.74 ± 17.77	85.90 ± 31.04	0.10
Mean percentage of compression time within all CPR time period (%)	59.06 ± 5.63	57.06 ± 7.98	0.26
Mean ventilation volume (mL)	404.61 ± 154.89	615.10 ± 449.57	0.018
Mean percentage of correct ventilation volume (%)	96.45 ± 17.99	75.10 ± 42.33	0.013

SD, standard deviation; bpm, beats per minute; CPR, cardiopulmonary resuscitation.

## Discussion

Our study confirmed that the self-instruction method was not inferior to instructor-led methods in overall performance at course conclusion. According to a systematic review of 22 randomised trials comparing the effect of self-instruction with that of traditional instruction in basic life support courses^24^, there were 12 studies including laypersons as the study participants^16–20,25–31^. The overall performance at course conclusion were measured in nine studies^16–20,25,28,29,31^. The participants in the self-instruction group had better performances in two of them^17,18^. The traditional instruction group was better in three studies^16,19,20^. The remaining four studies revealed that self-instruction and traditional instruction methods had similar educational effect on learners^25,28,29,31^, which is consistent with our study results. One study found that participants in self-instruction group retained skills longer^30^. However, three studies showed self-instruction and traditional instruction methods were similar in skill retention^28,29,31^. The above-mentioned studies provided evidence that a well-designed self-instruction method to teach CPR and AED could have the chance to reach the same as, at least no inferior to, the traditional instruction method. Alternative learning method such as E-learning had led to significantly reduced cost^32^. Self-instruction theoretically leads to further cost-saving, and more participants can learn the skills at the same time. The instructor-led method in our study is slightly different from the traditional course in the literature because we used a video teaching clip. However, it was found that trainees in digital resuscitation with online or video methods had no difference in knowledge score performance. Additionally, the self-instruction method may be suitable for some students who are not willing to join the traditional instruction course, such as those who have performance anxiety in front of people. Therefore, to increase the number of trained individuals as much as possible, different kinds of effective courses should be provided for the learners to choose from to meet their personal needs.

Our study revealed that the self-instruction group had better performance in compression-related skills, including compression depth, and mean percentage of full chest recoils. Our participants received two different types of feedback: automated feedback by the Resusci Anne QCPR manikin in the self-instruction group and instructors in the instructor-led group. The former was able to provide consistent compression quality feedback, while the latter made suggestions based on inspection and own experiences. It was reported that instructors’ judgments alone were insufficient, as 55% of inadequate compression depth was rated adequate, and approximately half of incorrect hand placement was rated adequate^33^. One prospective observational study also indicated that assessment by instructors in chest compression rate, depth, and fraction had poor sensitivity and specificity when compared to the data from the simulation manikin^34^. In one cluster randomised trial, trainees with the QCPR classroom–based instructor’s real-time objective feedback significantly increased the overall CPR score compared with conventional feedback^35^. Another study also found that students who received feedback from both manikin and instructor during hands-on practice sessions demonstrated significantly better BLS skills than those who only received feedback from an instructor^36^. Automated feedback by machine could overcome the disadvantage of instructor’s inaccurate feedback and it should be encouraged to add machine feedback into the instructor-led course during hands-on practice to improve the effectiveness of the training course. The 2020 ILCOR guideline also suggests the use of feedback devices that provide directive feedback on skill performance including compression rate, depth, recoil, and hand position during training courses^37^.

Regarding self-instruction or traditional instruction, previous systematic reviews did not draw conclusions about which one was superior^15,24^, whereas the convenience and lower expenditure of self-instruction seemed to be promising. In our study, although both groups had the same pass rate at course conclusion, participants in the self-instruction group had worse performance in confirming environmental safety and checking normal breathing < 10 s before starting CPR, which cannot be reminded by manikin feedback. A previous study had similar results significantly showing that the E-learning group performed the skill of ensuring safety worse than the instructor-led training groups^25^. These studies hinted at two directions for developing future educational courses for CPR and AED. First, for developing self-instruction training in the future, it is important to place more emphasis on the skills that could not be provided by manikin in the teaching material, such as confirmation of environmental safety and checking of normal breathing. Second, it may be beneficial to build blended learning with a combination of video teaching material and short-duration face-to-face instruction. In one study, novice providers in the self-directed group received an instructional kit and 90 min simulation session with an instructor before final skill assessment. It revealed no significant difference in skill performance between the self-instruction group and traditional training for the Neonatal Resuscitation Program^38^. Further, as psychological factors play a pivotal role in teaching and learning CPR^39^, the role of instructors not only provides skill feedback, but also plays as a motivation and enthusiasm facilitator^40^. A blended training course may be an alternative method for further resuscitation training.

Our study had some limitations. First, we did not include clinical outcomes of patients with cardiac arrest, such as survival to hospital discharge and neurologically intact survival. Second, we performed skill testing of one rescuer with a short duration. Nevertheless, in the real situation, there are usually several rescuers on the scene. Therefore, the learners may have different performances when they cooperate with others in the real situation. Third, the participants in our study were not blinded to the intervention. However, it was difficult to make participants blinded to the interventions in an educational study. Fourth, the participants were recruited among non-health workers via email invitations. They may be more interested in healthcare compared to the average population of laypersons and, therefore, had better performance. Nevertheless, they were randomly allocated and had similar background characteristics. It may reduce some bias. Fifth, we only performed non-inferiority trial and compared the difference between two types of teaching methods; therefore, the actual effect of self-instruction cannot be inferred in isolation. Finally, we did not perform retention testing after one specific period after training. More studies are needed to evaluate the effect on skill retention.

In conclusion, layperson CPR training using self-instruction with manikin feedback was not inferior to the instructor-led course in pass rate, and even better in some skill performances, whereas confirmation of environmental safety and check of normal breathing < 10 s before starting CPR should be emphasized in self-instruction training in the future.

## Supplementary information

Supplementary Information.
