# Supplementary material for: A non-inferiority randomised controlled trial comparing self-instruction with instructor-led method in training of layperson cardiopulmonary resuscitation
Source: Sci Rep. 2021 Jan 13;11:991. doi: 10.1038/s41598-020-79626-y (PMC7807060; doi:10.1038/s41598-020-79626-y)
Supplement: Supplementary file 1 — Supplementary Information. [file 41598_2020_79626_MOESM1_ESM.docx]

Supplementary Table 1. Skills checklist

| **Name： No.：**  **Place： Date： (DD/MM/YYYY) Evaluator：** | | | | |
| --- | --- | --- | --- | --- |
| **Individual Skill Items** | **Done**  **or performed most (>75%) of time** | **Performed often** | **Undone or**  **performed less (<25%) of time** | **Reason** |
| 1. Makes sure safety of the environment |  | XXX |  |  |
| 2. Checks consciousness |  | XXX |  |  |
| 3. Checks normal breathing < 10 sec |  | XXX |  |  |
| 4. Calls for help |  | XXX |  |  |
| 5. Corrects compression position |  | XXX |  |  |
| 6. Corrects compression rate (100–120 bpm) |  |  |  |  |
| 7. Corrects compression depth (5-6 cm) |  |  |  |  |
| 8. Completes chest recoil |  |  |  |  |
| 9. Opens airway |  |  |  |  |
| 10. Chest elevation when giving breaths |  |  |  |  |
| 11. Correct compression/breath ratio (30:2) |  | XXX |  |  |
| 12. No unnecessary compression interruptions |  |  |  |  |
| 13. Activates AED as soon as possible |  | XXX |  |  |
| 14. Corrects AED pad position |  | XXX |  |  |
| 15. Connects pad to AED machine |  | XXX |  |  |
| 16. Clears site when analysing rhythm by AED |  | XXX |  |  |
| 17. Clears site when defibrillation by AED |  | XXX |  |  |
| 18. Resumes chest compression immediately after defibrillation |  | XXX |  |  |

**Results: □ incompetent □ competent**
